# Biometric Data Comparison Between Lewis and Sprague Dawley Rats

**DOI:** 10.3389/fvets.2019.00469

**Published:** 2019-12-20

**Authors:** Richard Steiner, Madhu Dhar, Stacy M. Stephenson, Steven Newby, Austin Bow, Alisha Pedersen, David E. Anderson

**Affiliations:** ^1^Veterinary Medical Center, College of Veterinary Medicine, University of Tennessee, Knoxville, Knoxville, TN, United States; ^2^Department of Surgery, University of Tennessee Medical Center, Knoxville, Knoxville, TN, United States

**Keywords:** pressure mat, biometric, rodent gait analysis, rodent species effect, defect model

## Abstract

**Introduction:** Pressure mapping systems are often used for indirect assessment of kinematic gait parameter differences after repair of critical peripheral nerve defects in small animal models. However, there does not appear to be any literature that studies the differences in normal gait pattern of Sprague Dawley rats compared to Lewis rats using a Tekscan VH4 pressure mat system. The purpose of this study is to assess the gait profile of Lewis and Sprague Dawley rats generated by Tekscan's VH4 system to detect similarities and/or differences in gait parameters involving both force and temporal variables.

**Materials and Methods:** The gait profile of 14 Lewis and 14 Sprague Dawley rats was recorded using a Tekscan VH4 pressure map system with two successful walks per animal and gait parameter data was normalized for mean variance between the two rodent strains.

**Results:** The results showed that temporal and normalized force parameters were not significantly different between the two types of rats. Maximum force, contact area, stride length, and adjusted pressure variables were significantly different between the two strains, likely attributed to the body size and weight differential between the strains. Variation in some of these parameters were considered due to differences in overall body size between the two strains, variations in gait kinematics between individual rodent subjects, and the limitations of the current experimental design.

**Conclusion**: For future *in vivo* models, either Sprague Dawley or Lewis rat strains would be acceptable animal models when comparing base-line gait profiles using the Tekscan VH4 pressure map system when assessing critical defect repairs of peripheral nerves.

## Introduction

Animal models allow for investigation of medical device performance in a physiological system similar to humans, prior to clinical trials. In order for a class II or III medical device to be considered for clinical evaluation in human trials, the biometric data comparing changes in gait profiles before and after intervention must be assessed when translating from an animal model (1–3). To quantitatively assess tissue repair in animal models, certain tools are used to measure the biometrics of locomotion (4–6). It is therefore important to fully understand the normal gait pattern for any animal used in studies where gait parameter data will be recorded for the purposes of assessing repair of implant performance as a prerequisite for approval to begin clinical trials in humans.

While the types of animal species used and the specific tissues investigated in critical size defect models has changed very little, the tools and techniques used to measure gait patterns and report quantitative data has markedly improved (7–9). This is due, in part, to the inclusion of novel technologies that can more precisely and accurately detect specific gait parameters with minimal background noise. In addition, the augmentation of previous kinematic instruments by combining different technologies to create new instruments capable of producing more extensive gait profiles will improve the precision and accuracy of gait measurements. Reporting of gait measurements have been improved by further practice of using exogenous gait biometric data from previous tissue repair studies as an acceptable control standard for quantifying implant performance *in vivo*. Rats are one of the most common animals used in critical defect models for preclinical testing of class II and III medical devices (10–12). As the instruments for measuring gait parameters continue to develop via new technologies, it is important that normal gait parameters of different strains of rats are determined to provide data upon which to plan experiments. It is also valuable to compare normal gait biometrics between different strains of rats that use similar gait assessment tools in order to evaluate differences between strains used for specific defect models. This is especially relevant for biometric tools that find significant differences in the gait parameters between different strains of rats. A more predictable gait pattern could have a greater advantage in determining the success or failure of tissue repair in specific critical defect models *in vivo*.

Recently, pressure sensing mat technology has been developed as a more accurate and precise tool for quantifying gait patterns (13–15). We utilized a commonly used pressure sensing mat tool to measure the kinematic and timing variables in both the forelimb and hindlimb of rats to quantify the repair of peripheral nerve damage. Lewis and Sprague Dawley rats are the most widely used animal models for assessing nerve regeneration in critical size nerve defects (16, 17). Lewis rats are more often used as the subjects in critical nerve defect models over other alternative rodent strains due to minimal occurrence of autotomy behavior after an extended period post-transection of the sciatic nerve gap (18). This is supported by previous studies that have highlighted the physiological behavior changes in different rodent strains after induction of critical size nerve defects (19, 20). While previous studies have reported normal kinematic data from these rat strains, there does not appear to be any literature that compares the normal gait pattern of Sprague Dawley rats to Lewis rats using a pressure mat system (21–24). In this study we will be comparing gait parameters between Sprague Dawley and Lewis rats. We hypothesized that the normal walking gait motion between the two strains would be similar.

## Methods

The study was approved by the Institutional Animal Care and Use Committee (IACUC) at the University of Tennessee, Knoxville (IACUC# 2574-0318).

### Pressure Sensing Mat Specifications

**Figure 1:**
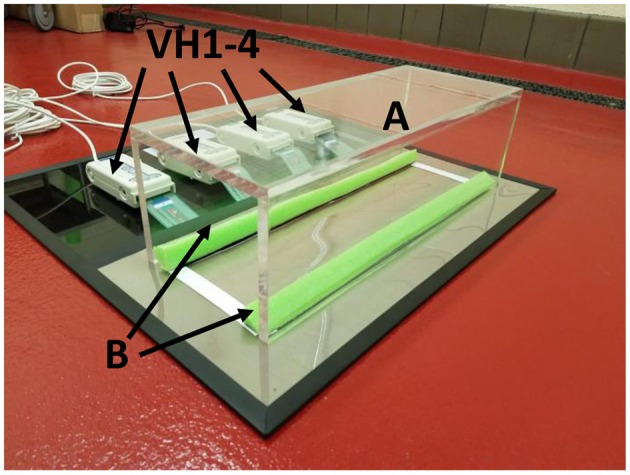
Tekscan VH4 pressure-mat system setup. Testing apparatus consists of a tinted Plexiglas tunnel **(A)**. The tunnel is positioned over the Tekscan pressure mat aligned to Styrofoam borders placed at the edges of the sensor area **(B)**. Four sensors (VH1–4) connect the four 5,101 grids to the Tekscan mat.

Gait patterns were analyzed using a pressure sensor mat (Tekscan VH4, Tekscan, Boston, MA). This mat is composed of four 5,101 high-resolution pressure sensor grids laid out side by side. Based on the current Tekscan manual, the sensing grid has rows and columns of sensors that converts a change in electrical impedance to a force based on the calibration of the mat to a known applied weight. Each sensor grid is composed of 44 columns and 44 rows (11.2 × 11.2 cm) of sensors. When laid out in tandem, the entire surface consists of 176 sensor columns × 44 sensor rows for a total area of 11.18 × 44.7 cm (499.75 cm^2^) with a 0.127-cm gap between each sensor. The total pressure grid has 7,744 sensors at a density of 15.5 sensors/cm^2^. The sensors were aligned to place the origin (0,0) in the upper left corner of the sensor grid (25). Studies that have utilized the VH4 system (Tekscan VH4, Tekscan, Boston, MA) have reported excellent calibration reliability of the 5101 sensors in *in vivo* studies of 1.2–4.4% pressure units, well within the established acceptable cut-off range of 5% (7). A previous study also reported this as an acceptable range when reporting sensor reliability ranges of 3–4% (26). For the purpose of this experiment, the gait testing unit was modified to include a tinted Plexiglas tunnel (width 17.0 cm, height 17.0 cm, length 44.7 cm) and a Styrofoam side wall (width 2.54 cm, height 2.54 cm, length 44.7 cm) for the purpose of guiding the rats across the mat and to insure that the rats remain on the sensor area during the rats walk across the mat. This minimized false data recordings from animal false-steps on the edges or outside the sensor matrix area (Figure 1).

The pressure sensing mat was calibrated using a phantom device designed to support a known mass (2,006.8 g; 4.42 lb.; 19.7 N). The instrument was programmed to record gait parameters after the operator manually selects to star recording at a sample rate of 50 frames/s and to automatically stop recording after 20 s unless manually stopped by the software operator. Calibration accuracy was tested by comparing five repeated measures of the phantom weight. Tekscan software records the raw data of each gait variable as an average mean value for each trial walk in an ASCII file format. Each limb must record at least 3 footfalls (“hits”) on the mat to calculate an average value, otherwise the software will record an N/A for the calculated variable.

**Figure 2:**
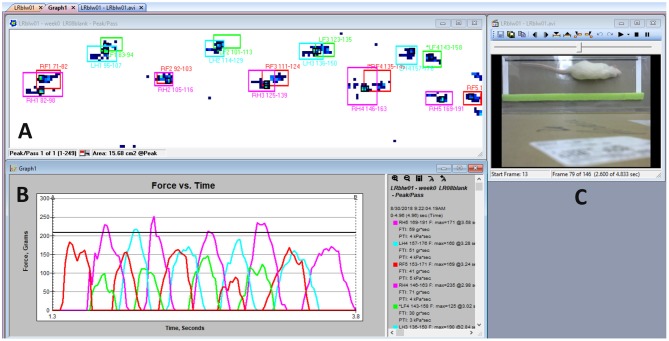
Tekscan software display. **(A)** Example gait force profile during the subjects walk from right to left. Visual display of right forelimb (red), left forelimb (green), right hindlimb (pink), and left hindlimb (blue) contact sensor boxes is represented as the summation of sensors activated and their corresponding limb recording locations on the mat, time, and gait variables. **(B)** Graph of force vs. time for the example gait profile of force peaks with their respective limbs. **(C)** Logitech camera visual output.

A Logitech C270 web video camera was attached to a support located approximately 2 feet directly above the center point of the sensor area path and synced to the Tekscan software gait profile to record alongside the pressure hits detected on the mat to verify a successful pass across the mat and to match the limbs with the corresponding limb strikes in the gait profile (not shown in Figure 1). The web video camera was synced to the Tekscan software program to record at the same rate and stop when the software recording stops. The operator used the video synchronization in order to identify and correct software errors in selecting the appropriate limb strike box or if the software was unable to discern multiple limb strikes at a single location due to ipsilateral forelimb and hindlimb contact which often overlap each other. This made it necessary to manually correct contact boxes for each limb in the software by following the subject's motion on both the camera and gait force profile for corrected gait measurements (Figure 2). The type of data output from pressure mat systems includes both temporal and force gait data. Temporal data analyzed in this study includes stance time (how long each limb makes contact with mat), swing time (the time it takes between two hits on the mat with the same hindlimb or forelimb), stride time (the time it takes between right forelimb and left hindlimb to make contact with the mat at the same time as well as the opposite limb pattern), stride length (distance between right forelimb and left hindlimb as well as its opposite limb pattern) stride velocity, stride acceleration, and limb surface area coverage. Force data includes the limb forces generated on the mat with respect to pressure difference, normalized force data to the body weight of each rodent, impulse force in each hindlimb and its normalized version to the rodent bodyweights, and the peak pressure generated in each hindlimb. The system is also capable of comparing specific limbs to each other as a ratio profile for maximum force generation, stance time, stride time, stride length, and stride velocity variables. These ratios concern 4 areas of comparison toward these variables: forelimbs to hindlimbs, all limbs on the right side of rodent to all limbs on the left side, right forelimb to left forelimb, and right hindlimb to left hindlimb.

### Gait Testing Protocol

Lewis and Sprague Dawley rats were selected for use in the study of gait biometrics. Male Sprague Dawley and Lewis rats (acquired from Envigo) arrived at their assigned holding location at approximately 8 weeks of age (*n* = 14, each) and allowed to acclimate to their environment for 1 week. During the acclimation period, rats were exposed to rodent handling procedures and the pressure mat system to improve rodent comfort with handling by study personnel and performing walks on the mat. During this period of acclimation in accordance with IACUC protocols, the rodents were observed on a daily basis by qualified veterinarians to assess the health and well-being of the rodents including several parameters directly related to movement. Those parameters included loss of appetite, restlessness/distress, lethargy, loss of mobility, failure to groom, self-mutilation/biting, and significant reduction in weight (>10%). Any assessment that was considered outside of normal parameters warranted removal of the test animal from the study.

After 1 week of acclimation, each animal was directed to walk two passes across the pressure mat during a single testing period. Gait measurements were subsequently taken at 9 weeks of age. During the acclimation period, none of the animal subject were removed from the study indicating that all animal subjects were healthy and were not orthopedically impaired or displayed any other condition that might influence normal movement on the day of testing.

Calibration of the pressure sensors was carried out before each use of the device. Each rat was weighed using a digital scale before starting walk trials on the mat. The rats would be released by the handler on either side of the pressure mat facing the open end of the transparent Plexiglas tunnel. Rats were positioned and held by the handler/operator's hand at either end of the tunnel openings and released when the test was initiated by manually starting the recording procedure in the software by the operator. Animals were enticed to walk across the mat by placing food/treats at opposite ends of the tunnel. The animals then traversed the length of the tunnel until they reached the other end, at which time the recording was stopped manually, and a successful or incomplete pass was determined by the software operator. The rodents were not controlled for their speed during the assessment and had ample room on the mat to follow a path that is not perfectly linear; it is therefore likely that mean gait parameters would vary considerably between runs within each rat.

Criteria for a successful walk across the mat included that the rats were able to walk continuously with uncontrolled velocity from one end of the sensor area of the mat to the other with minimal to no pause in gait motion, and at least three strikes from each limb was detected by the software and confirmed by reviewing the video recording and the measured biometric data. If the gait of the rodents resulted in failure of any of these criteria, the walk was considered unsuccessful, at which point the software data and video recording was reset and the rodents were repositioned at either end of the mat to begin another pass. All data recorded for the gait parameters were average values from each successful trial. Data was reported as the average peak measures of each gait variable and for each limb as recorded by the sensors and identified as either the right forelimb (RF), left forelimb (LF), right hindlimb (RH), or left hindlimb (LH) for the trial.

Gait temporal variables measured included average stance time, swing time, stride time, stride length, stride velocity, and stride acceleration based on the location of the limb strikes relative to the sensor area and other subsequent limb strikes. Contact variables included contact area derived from the maximum number of sensors loaded. Force generation parameters included contact force, impulse force, and contact pressure derived from the maximum sensor readings at the time of impact. Contact pressure, impulse force and contact force were normalized to the weight of the animals on the date of testing (average value divided by body weight). Time variables (stance time, stride time, stride length, and stride velocity) and maximum force were normalized to specific limb orientations (forelimbs to hindlimbs, left side limbs to the right sided limbs, left forelimb to right forelimb, and left hindlimb to right hindlimb).

### Statistical Analysis

All data from successful trials were analyzed by SAS software (STAT 12.1). Mean gait parameter data compared by rodent strain was analyzed as a paired *t*-test for significance of mean difference. Two-way ANOVA was assessed by comparing gait variables to rodent stains based on limbs followed by *post-hoc* Tukey's test of mean grouping. Significance was set at *P* ≤ 0.05.

## Results

### Normal Gait Assessment

**Figure 3:**
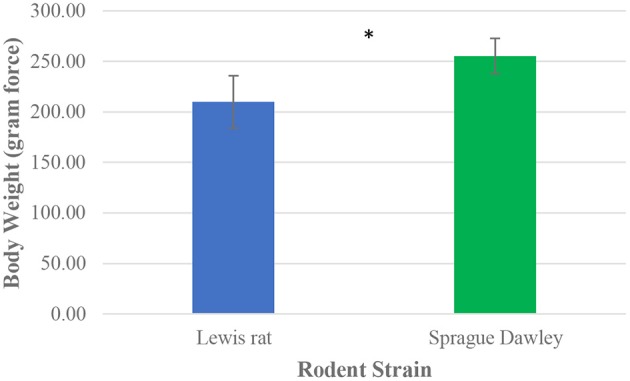
Mean body weight distribution between Lewis and Sprague Dawley rats. Paired *t*-test analysis. *P ≤ 0.05.

All 28 rodent subjects from the Sprague Dawley (*n* = 14) and Lewis (*n* = 14) rat groups completed 2 successful walk trials. This resulted in a total of 28 successful gait trials per group, giving a total of 56 data sets of mean values of gait parameters. A range of 4–10 unsuccessful trials was observed per rat tested before obtaining 2 successful trials to be included in gait analysis. Paired *t*-test detected significant differences in the initial body weight of the rat groups, with the Sprague Dawley rat group having a mean body weight 26.1% greater than the Lewis rat group (Figure 3).

### Gait Analysis: Ratio Temporal Parameters

**Figure 4:**
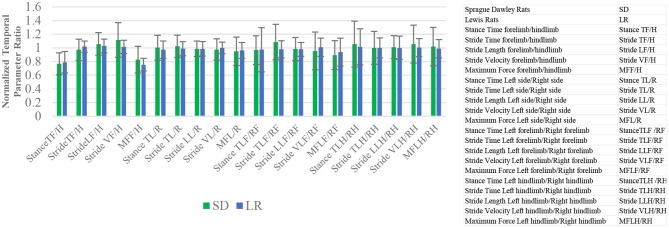
Mean and standard deviation ratio temporal and force parameter values. Stance time, stride time, stride length, stride velocity, and maximum force comparative analysis based on limbs. The closer the mean values are to 1.0, the less variance there is between limbs of the animals. Paired *t*-test analysis. *P ≤ 0.05.

The ratio mean values indicate the degree of variance between different limbs for specific temporal variables (Figure 4). Paired *t*-test analysis showed no significant difference in any of the ratio configurations of normalized temporal parameter ratios to rodent limbs based on rodent strains.

Mean gait data and standard deviations for all parameters comparing rodent strain and limbs are categorized in Tables 1–3.

**Table 1:** Ratio values for normalized temporal parameters.

**Strain**	**Stance TF/H**	**Stride TF/H**	**Stride LF/H**	**Stride VF/H**	**MFF/H**
SD	0.77 ± 0.2	0.97 ± 0.2	1.06 ± 0.2	1.12 ± 0.3	0.83 ± 0.2
LR	0.79 ± 0.2	1.02 ± 0.1	1.03 ± 0.1	1.02 ± 0.1	0.76 ± 0.1
**Strain**	**Stance TL/R**	**Stride TL/R**	**Stride LL/R**	**Stride VL/R**	**MFL/R**
SD	1.00 ± 0.2	1.02 ± 0.2	0.99 ± 0.1	0.98 ± 0.2	0.95 ± 0.2
LR	0.97 ± 0.1	0.99 ± 0.1	0.99 ± 0.1	1.0 ± 0.1	0.96 ± 0.1
**Strain**	**Stance TLF/RF**	**Stride TLF/RF**	**Stride LLF/RF**	**Stride VLF/RF**	**MFLF/RF**
SD	0.97 ± 0.2	1.08 ± 0.3	0.98 ± 0.2	0.96 ± 0.3	0.89 ± 0.2
LR	0.97 ± 0.3	0.98 ± 0.1	0.98 ± 0.1	1.01 ± 0.1	0.94 ± 0.2
**Strain**	**Stance TLH/RH**	**Stride TLH/RH**	**Stride LLH/RH**	**Stride VLH/RH**	**MFLH/RH**
SD	1.06 ± 0.3	1.00 ± 0.2	1.01 ± 0.2	1.06 ± 0.3	1.02 ± 0.3
LR	1.02 ± 0.3	1.00 ± 0.2	1.00 ± 0.2	1.00 ± 0.1	0.99 ± 0.1

**Table 2:** Values for # of stance, gait time, gait distance, gait velocity, gait cycle time, and cycle min.

**Strain**	**No stance**	**Gait time (sec)**	**Gait distance (cm)**	**Gait velocity (cm/sec)**	**Gait cycle time** **(sec)**	**Cycles min** **(sec)**
SD	14.43 ± 2.3	1.88 ± 0.9	30.47 ± 5.6	19.85 ± 9.8	0.62 ± 0.2	110.43 ± 39.5
LR	17.93 ± 1.9	2.04 ± 0.6	37.32 ± 3.7	19.85 ± 6.2	0.50 ± 0.1	125.30 ± 25.5

Table 3Values based on specific limbs for both strains.

**Table:** 

**Strain**	**Extremity**	**Stance time** **(sec)**	**Swing time (sec)**	**Stride time (sec)**	**Stride length (cm)**	**Stride velocity (cm/sec)**	**Stride Acc (cm/sec^**2**^)**
LR	LF	0.30 ± 0.1	0.22 ± 0.1	0.49 ± 0.1	9.37 ± 1.4	20.27 ± 6.4	−2.05 ± 26.9
	LH	0.40 ± 0.2	0.13 ± 0.1	0.49 ± 0.1	9.22 ± 1.5	19.80 ± 5.6	2.81 ± 36.6
	RF	0.32 ± 0.1	0.21 ± 0.1	0.51 ± 0.1	9.62 ± 1.3	20.21 ± 6.3	1.15 ± 24.4
	RH	0.41 ± 0.2	0.14 ± 0.2	0.49 ± 0.1	9.34 ± 1.4	19.88 ± 5.6	14.80 ± 35.9
SD	LF	0.36 ± 0.1	0.30 ± 0.2	0.63 ± 0.2	10.58 ± 2.3	19.73 ± 9.6	5.93 ± 25.3
	LH	0.50 ± 0.2	0.18 ± 0.1	0.63 ± 0.3	10.54 ± 2.3	19.30 ± 8.1	−8.95 ± 20.7
	RF	0.37 ± 0.1	0.25 ± 0.2	0.60 ± 0.3	10.96 ± 2.2	21.73 ± 10.4	5.12 ± 32.5
	RH	0.49 ± 0.2	0.18 ± 0.1	0.65 ± 0.3	10.33 ± 1.9	18.94 ± 10.0	13.4 ± 35.9

**Table:** 

**Strain**	**Extremity**	**MF BW** **(%)**	**MF** **(gr)**	**Impulse BW (%)**	**Impulse** **(gr)**	**Max Pressure** **(kPa)**	**Contact Area (cm**^**2**^**)**	**Adj Pressure (g/cm**^**2**^**)/BW**
LR	LF	61.93 ± 10.5	133.90 ± 29.8	10.86 ± 3.1	23.64 ± 8.3	47.70 ± 9.7	0.51 ± 0.1	1.30 ± 0.4
	LH	84.71 ± 9.7	183.60 ± 37.2	20.09 ± 7.9	44.46 ± 22.5	55.53 ± 9.7	0.61 ± 0.1	1.46 ± 0.3
	RF	67.02 ± 10.6	144.30 ± 26.8	12.20 ± 3.0	26.57 ± 8.5	49.04 ± 7.9	0.52 ± 0.1	1.35 ± 0.2
	RH	86.55 ± 10.5	187.30 ± 35.3	20.50 ± 6.3	44.80 ± 17.1	56.20 ± 10.1	0.63 ± 0.1	1.44 ± 0.3
SD	LF	63.64 ± 13.2	161.0 ± 33.9	12.70 ± 4.5	31.93 ± 10.7	51.75 ± 9.6	0.57 ± 0.1	1.13 ± 0.2
	LH	84.48 ± 15.0	215.90 ± 43.1	24.57 ± 9.5	62.96 ± 25.7	61.40 ± 7.0	0.70 ± 0.1	1.22 ± 0.2
	RF	74.83 ± 19.3	189.80 ± 45.0	14.18 ± 5.5	35.86 ± 12.7	56.46 ± 10.0	0.61 ± 0.1	1.23 ± 0.2
	RH	85.29 ± 16.6	217.90 ± 46.1	24.60 ± 12.1	63.11 ± 32.7	64.07 ± 8.5	0.73 ± 0.1	1.19 ± 0.2

### Gait Analysis: Force and Temporal Parameters

**Figure 5:**
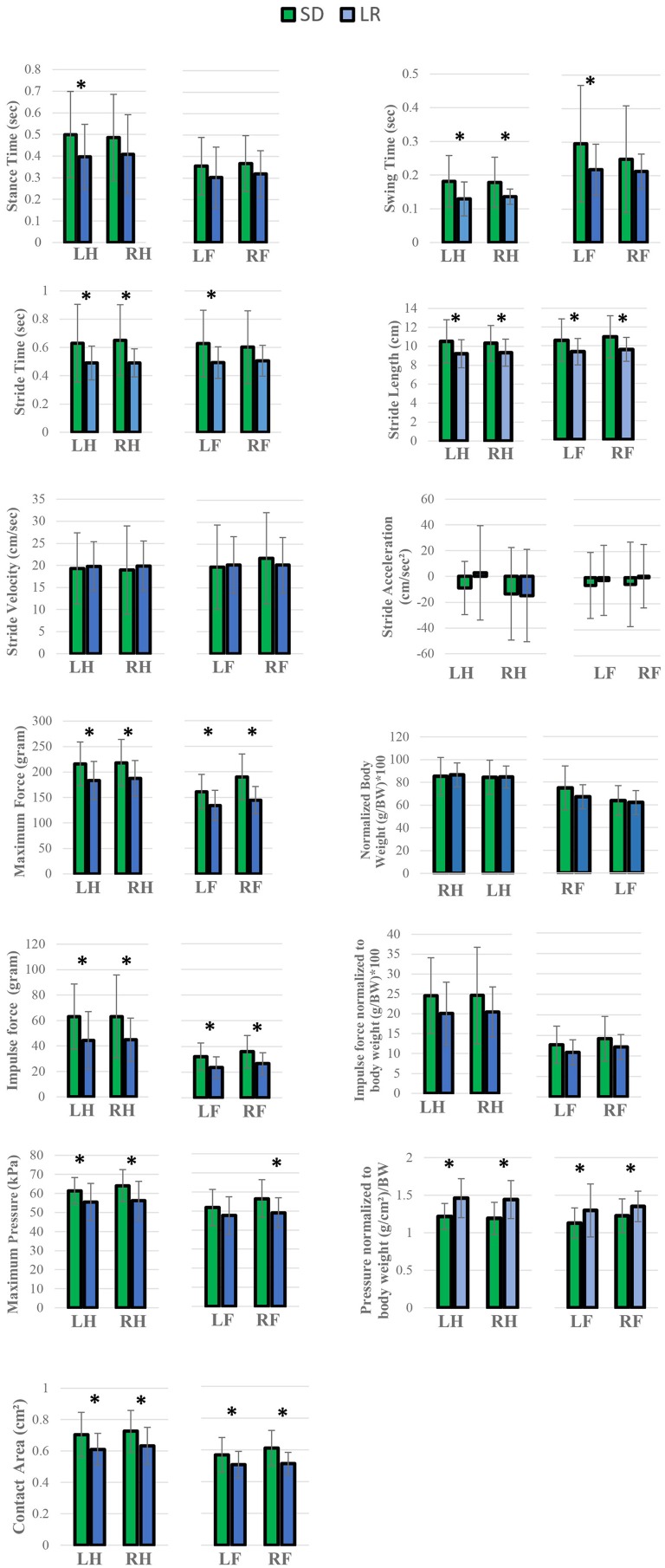
Analysis of variance of gait variables. Two-way ANOVA *P < 0.05 analysis. Comparison of the mean values between Lewis Rats (LR) and Sprague Dawley rats (SD), subcategorized by left forelimb (LF), right forelimb (RF), and left hindlimb (LH), and right hindlimb (RH). All values are presented as group means and SD error bars. *P < 0.05.

**Figure 6:**
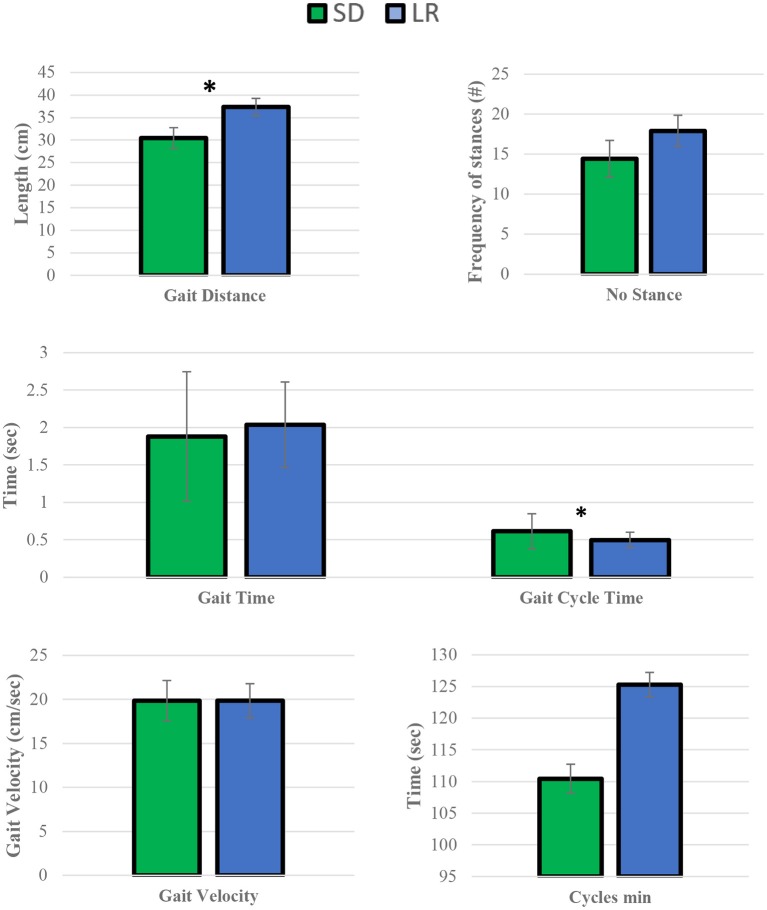
Mean temporal parameter values. Sprague Dawley (SD) and Lewis (LR) rats paired *t*-test. *P ≤ 0.05.

Force and temporal parameter values specific to the four limbs that make up the gait profile of each animal group were recorded and are presented in Figures 5, 6. Two-way ANOVA analysis showed that there were no significant differences for both the Sprague Dawley and Lewis rat groups in stride velocity and stride acceleration temporal parameters for each limb. The analysis also determined that the normalized maximum and normalized impulse forces in the groups for each limb were not statistically different. Stride length, impulse force, maximum force, contact area, and normalized pressure showed significant differences between the groups for each limb tested. Swing time, and stride time showed significant differences between the groups for the hindlimbs and the left forelimb while the right forelimb showed no significant difference. The stance time showed a significant difference for the left hindlimb only. The maximum peak force showed significant differences for all limbs except the left forelimb.

Gait parameters recorded from left and right hind limbs from each rat group were also measured for mean variance grouping using *post-hoc* Tukey's test to determine how similar the mean force and temporal values were for each limb. In the Lewis group stride time, stride length, stride velocity, stride acceleration, and normalized pressure in each limb were found to be statistically similar to each other. Swing time, maximum force, normalized maximum force, impulse force, normalized impulse force, and contact area were statistically different between the forelimbs and the hindlimbs with the hindlimb mean values having a higher value compared to the forelimb. Maximum pressure and stance time mean variables for the Lewis group limbs were grouped based on their statistical mean variance from each other.

Similar to the Lewis group, the Sprague Dawley group under *post-hoc* Tukey's test showed stride time, stride length, stride velocity, stride acceleration, and normalized pressure in each limb were found to be statistically similar. Stance time, impulse force, normalized impulse force, and contact area were statistically different between the forelimbs and the hindlimbs with the hindlimb mean values having a higher value compared to the forelimb. Swing time, maximum pressure, maximum force, and normalized force mean variables for the Sprague Dawley group limbs were grouped based on their statistical mean variance from each other.

Paried *t*-test of the mean temporal parameters for gait distance and gait cycle time were found to be significantly different between the two strains, while all other remaining variables (cycle min, gait velocity, gait time, and number stance) were found to not be significantly different between the two groups.

We have identified both similarities and differences in the gait kinematics between Sprague Dawley and Lewis strains. Large variances (*P* < 0.05) were detected in both the Sprague Dawley and the Lewis rat groups when comparing the forelimbs to the hindlimbs for maximum force and stance time variables. All other limb groups and variables showed mean values with variances that were not significant, indicating small differences in gait temporal values between the limbs of the rats. The results of paired *t*-test of mean gait parameters between both rodent strains these results are presented in Table 4. Variables for speed, acceleration and normalized forces generated in each limb of the Sprague Dawley and the Lewis rat groups had similar values and variations given the normal conditions of the rats studied from each strain. The analysis also showed differences between unadjusted impulse force, maximum force, contact area, stride length, and adjusted pressure values detected in each limb in both strains of rats. The temporal parameters of stance time, swing time, and stride time indicated similar swing and stride times for the right forelimb yet significant difference in the stance time for the left hind limb between the two groups.

Table 4Mean comparison between Sprague Dawley and Lewis groups based on limbs.

**Table:** 

**Strain**	**Stance time (sec)**	**Swing time (sec)**	**Stride time (sec)**	**Stride length(cm)**	**Stride velocity (cm/sec)**	**Stride acceleration (cm/sec^**2**^)**
LF	0	1	1	1	0	0
LH	1	1	1	1	0	0
RF	0	0	0	1	0	0
RH	0	1	1	1	0	0

**Table:** 

**Strain**	**MF BW (%)**	**MF** **(gr)**	**Impulse BW** **(%)**	**Impulse** **(gr)**	**Max peak pressure** **(Pa)**	**Contact area (cm**^**2**^**)**	**Adj pressure** **(g/cm**^**2**^**)/BW**
LF	0	1	0	1	0	1	1
LH	0	1	0	1	1	1	1
RF	0	1	0	1	1	1	1
RH	0	1	0	1	1	1	1

*Sprague Dawley and Lewis rats, subcategorized by left forelimb (LF), right forelimb (RF), and left hindlimb (LH), and right hindlimb (RH). Two-way ANOVA: 1 = (P < 0.05), 0 = (P ≥ 0.05)*.

Tukey's test applied to the mean values of the limbs from each group (Tables 5, 6) showed that swing and stance times had different mean groupings between the limbs for each strain indicating variable locomotion in the normal gait of these rats.

Table 5Lewis mean value group comparison between limbs.

**Table:** 

**Lewis**	**Stance time (sec)**	**Swing time (sec)**	**Stride time (sec)**	**Stride length (cm)**	**Stride velocity (cm/sec)**	**Stride acceleration (cm/sec^2^)**
LF	1	1	0	0	0	0
LH	1, 2	2	0	0	0	0
RF	1, 2	1	0	0	0	0
RH	2	2	0	0	0	0

**Table:** 

**Lewis**	**MF_BW (%)**	**MF** **(gr)**	**Impulse BW (%)**	**Impulse (gr)**	**Max peak pressure** **(Pa)**	**Contact area (cm**^**2**^**)**	**Adj pressure (g/cm**^**2**^**)/BW**
LF	1	1	1	1	1	1	0
LH	2	2	2	2	2, 3	2	0
RF	1	1	1	1	1, 2	1	0
RH	2	2	2	2	3	2	0

*Post-hoc Tukey's test: RF, right forelimb; LH, left hindlimb; RH, right hindlimb. 1, 2, 3 = individual groups for mean variance, 0 = similar mean variance group*.

Table 6Sprague Dawley mean value group comparison between limbs.

**Table:** 

**Sprague Dawley**	**Stance time (sec)**	**Swing time (sec)**	**Stride time (sec)**	**Stride length (cm)**	**Stride velocity (cm/sec)**	**Stride acceleration (cm/sec^**2**^)**
LF	1	1	0	0	0	0
LH	2	2	0	0	0	0
RF	1	1, 2	0	0	0	0
RH	2	2	0	0	0	0

**Table:** 

**Sprague Dawley**	**MF BW (%)**	**MF (gr)**	**Impulse BW (%)**	**Impulse (gr)**	**Max peak pressure (Pa)**	**Contact area (cm**^**2**^**)**	**Adj pressure (g/cm**^**2**^**)/BW**
LF	1	1	1	1	1	1	0
LH	2	2	2	2	2, 3	2	0
RF	1, 2	1, 2	1	1	1, 2	1	0
RH	2	2	2	2	3	2	0

*Post-hoc Tukey's test: RF, right forelimb; LH, left hindlimb; RH, right hindlimb. 1, 2, 3 = individual groups for mean variance, 0 = similar mean variance group*.

## Discussion

Our analysis of the control group data suggests that for the purposes of collecting gait biometric data as a quantitative measurement of tissue repair in critical size defect models, the use of either rat strain is acceptable as a positive gait control model. To our knowledge, this is the first study to evaluate the gait pattern between Sprague Dawley and Lewis rat strains using a pressure mapping system. Differences between the two groups could be attributed to rats with gait patterns that favor a particular limb over the others, creating uneven gait kinematics and locomotion during normal gait motion. Thus, while this system mimics natural locomotion behavior more closely, this system is not able to ensure the animals will move at the same speed. Each individual rodent subject has a degree of intraindividual and interindividual variability that has been reported in studies that use pressure mats since these instruments are incapable of controlling for these factors that could significantly impact data output (27, 28). This has been suggested from previous studies that have noted similar observations in the normal gait pattern of rodents (7, 29).

Forward motion variables such as velocity and acceleration were consistent between both groups, including the additional analysis of individual limbs regarding these variables. This would indicate that the motion of each of the limbs of the rats was the same for both groups. Due to the difference in animal size between the groups, swing time and stride length variables were different between the two groups, where the Sprague Dawley group showed greater stride length and swing time than the Lewis group which has been reported previously (27). This also explains why the Sprague Dawley group had on average a lower mean value of stance number, gait length, and gait time compared to the Lewis group. Normalized maximum and impulse forces were consistent between both groups as indicated by the force generated in each limb during both deceleration and acceleration of the limbs on the mat were the same between both groups when normalized to the weight distribution of the rats in each of their respective limbs. This was further confirmed when grouping the mean values of the limbs for each group, which showed statistically similar stride velocity and acceleration for each limb while normalized force and contact area values remained statistically different between forelimbs and hindlimbs. Previous studies have used rodents as a control group for normal gait motion and have described this gait pattern where the hindlimbs will support more of the rodent's weight compared to the forelimbs due to unequal distribution of the rodent's weight across its body frame from the front end to the back end (7, 23). This also explains why the stance time variable has a large variance between the forelimbs and hindlimbs for both rodent groups, as the greater weight supported by the hindlimbs needs more time to stabilize and propel the body forward during normal gait motion. Thus, during normal gait motion in rodents the hindlimbs are considered more responsible for producing forward motion, while the forelimbs are more concerned with directing forward motion in the intended direction.

Our results show that for normalized force and stride velocity/acceleration variables, both groups maintained similar weight distributions and forward motion in each limb during normal walk patterns. Unadjusted variables for contact area, force and stride length showed significant differences between the two groups for each hind and fore limb from each group. We also detected irregular patterns in the swing, stride, and stance time variables between the two groups, which we attribute to uneven gait motion during the experiment due to favoring or odd sensation from one or more limbs.

Certain limitations to pressure mapping systems should be considered such as rodent biometric specifications that could have a larger impact on normal rodent gait patterns such as male vs. female gait patterns. Additional factors to consider are other rodent strains incorporated into the study and different weight to size ratios caused by age differences in skeletally immature frames (8–14 weeks old) to skeletally mature frames (15 weeks or older) (30). Environmental factors such as time and form of training and conditioning prior to testing, ease of housing and handling different rodent strains, and potential physiological changes brought on by stress or pathogens. Comparison of the Tekscan VH4 model results with other highly sensitive pressure mat systems results might be prudent to help establish control rodent gait patterns for other commonly utilized gait assessment tools. Trial runs per rodent for this study were selected to minimize variability caused by animal behavior changes and stress from increased animal handling, hence the decision to test with only two successful trials. It is suggested that future studies to assess gaits with pressure mats could minimize variability between independent rodent subjects by either controlling more for rodent speed and guidance during gait walks across the mat or by increasing the number of successful trials per rodent. Lastly, it is important to address the limitations of the instruments to accurately sense and record gait parameters. Events occurring with the equipment that could lead to false data interpretation include incorrect calibration (standard error of 5% is referenced), bedding, feces or urine on the mat, or sensor contact with the plastic cover not caused by a limb hit point. Without controlling for these potential errors, false data will provide misinterpretation of actual rodent gait profiles and suggest similarities or differences in gait parameters were no such relationship exists. This is possible by detecting false negative differences in strains of similar weight and size (type II error) and false positive differences in strains of very different weight and size (type I error). As suggested by controlling for variability of gait parameters of single rodent subjects between trials, we can minimize statistical errors and ensure accuracy of instrument readings by increasing the number of trials per rodent and controlling for artifacts that could provide false data.

## Conclusion

To conduct research on peripheral nerve critical defect models, we have used the VH4 Tekscan pressure mapping gait system to establish a control standard gait model and provide valuable animal gait biometric data to differentiate gait patterns between Sprague Dawley and Lewis rat strains. This data is to further assist our group in differentiating gait patterns in these strains after induced peripheral nerve defects as a measure of nerve repair. The statistical similarities between temporal and normalized force parameters between the two strains indicate that for the purposes of collecting gait biometric data as a quantitative measurement of tissue repair in critical size defect models, either Sprague Dawley or Lewis rat normal gait patterns can be used interchangeably as acceptable positive gait control models. This system has also proved useful in selecting appropriate animals for critical defect models where animal gait biometric data is relevant and the tool for measuring gait patterns requires a pressure mapping gait system. We have shown for the first time, comparative quantitative assessment of normal gait patterns in Sprague Dawley to Lewis rats highlighting specific similarities and differences in gait patterns. This data would suggest that the gait pattern for Sprague Dawley rats are similar to that of the gait pattern of Lewis rats when using Tekscan' s VH4 model pressure mat system and that in terms of establishing a base-gait profile either animal strain is an acceptable model for the assessment of a variety of critical defect repairs.

## Data Availability Statement

All datasets generated for this study are included in the article/supplementary material.

## Ethics Statement

This animal study was reviewed and approved by University of Tennessee, Knoxville (IACUC# 2574-0318).

## Author Contributions

All authors listed have made a substantial, direct and intellectual contribution to the work, and approved it for publication.

### Conflict of Interest

The authors declare that the research was conducted in the absence of any commercial or financial relationships that could be construed as a potential conflict of interest.
